# An In Vitro SEM Study on the Effectiveness of Smear Layer Removal of Four Different Irrigations

**Published:** 2012-10-13

**Authors:** Syed Mukhtar-Un-Nisar Andrabi, Ashok Kumar, Rajendra Kumar Tewari, Surrendra Kumar Mishra, Huma Iftekhar

**Affiliations:** 1. Department Of Conservative Dentistry and Endodontics, Dr. Z.A. Dental College, Aligarh Muslim University, Aligarh, India.

**Keywords:** Smear Layer, EDTA, MTAD, Endodontic, Irrigation, Scanning Electron Microscopy

## Abstract

**Introduction:**

The aim of this study was to compare the smear layer removal efficacies of 3% sodium hypochlorite (NaOCl), 17% Ethylenediaminetetraacetic acid (EDTA), SmearClear and BioPure MTAD using a common irrigation protocol.

**Materials and Methods:**

Fifty freshly extracted human single rooted maxillary and mandibular teeth were prepared by a ProTaper rotary system up to an apical preparation file size F3. Prepared teeth were randomly divided into five groups (n=10); distilled water (Group A; negative control), EDTA (Group B), SmearClear (Group C), BioPure MTAD (Group D) and NaOCl (Group E). After final irrigation with tested irrigants the teeth were decoronated, split into two halves longitudinally and observed under a scanning electron microscope (SEM) for removal of the smear layer. The SEM images were then analyzed for the amount of smear layer present using a three score system. Data were analyzed using the Kruskal-Wallis test and Mann-Whitney U test.

**Results:**

Intergroup comparison of groups B, C, and D showed no statistical significant differences in the coronal and middle thirds, however, in the apical third the canal surfaces were cleaner in samples from group D (P<0.05).

**Conclusion:**

BioPure MTAD was the most effective agent for the purpose of smear layer removal in the apical third of the root canals.

## Introduction

The success of endodontic therapy relies on proper biomechanical preparation, disinfection and three-dimensional obturation of the root canal system. During cleaning and shaping of the root canal system, mechanical instrumentation leaves a smear layer covering the dentinal walls [1][2][3]. The first researchers to describe the smear layer on the surface of instrumented root canals were McComb and Smith [1]. They suggested that the smear layer consisted not only of dentin, as in the coronal smear layer, but also the remnants of odontoblastic processes, pulp tissue and bacteria. Mader et al. reported that the smear layer thickness was generally 1-2 µm [3]. They discussed the smear material in two parts: first, the superficial smear layer, and second, the material packed into the dentinal tubules. Packing of smear debris was present in the tubules to a depth of 40 µm. The smear layer, being a loosely adherent structure, should be completely removed from the surface of the root canal wall because it can harbor bacteria and provide an avenue for leakage [4][5][6]. The smear layer can limit the disinfecting action of intracanal medicaments and also act as a barrier between filling materials and the canal wall and therefore compromise the formation of a satisfactory seal [7][8][9][10][11].

Literature currently available supports the removal of the smear layer for improved disinfection and better adaptation of materials to the canal walls [6][7][8][11].

Various methods of smear layer removal include chemical [12][13][14][15], ultrasonic [16] and laser techniques [17], none of which are totally effective or are used universally [6][18].

So far the most commonly used method of smear layer removal has been the chemical method using chelating agents, with EDTA being the most common agent used [18]. EDTA has been tested in different concentrations, in different formulations and for different time periods [18][19][20]. The most advocated combination of 17% EDTA plus 5.25% NaOCl removes the smear layer completely in the coronal and middle thirds but is less effective in the apical third. Other disadvantages of EDTA are dentinal erosion in the coronal and middle third of root dentin and its limited antibacterial activity [19][20].

SmearClear (Sybron Endo, Orange, CA) a recently introduced product for the purpose of removing the smear layer is a solution of 17% EDTA with a cationic (cetrimide) and an anionic surfactant.

The introduction of MTAD (Dentsply, Tulsa Dental, Tulsa, OK, USA) (mixture of tetracycline, acid and detergent) represents an advance in endodontic irrigation research. MTAD has been reported to remove the smear layer effectively, eliminate microbes that are resistant to conventional endodontic irrigants and dressings, and provide sustained antimicrobial activity.

There is no single advocated irrigation protocol that dictates volume, time of exposure or the manner in which the irrigant is delivered to the canal to achieve optimal results.

This study aimed to compare the smear layer removal efficacies of the following four different solutions using a single common irrigation protocol: 3% sodium hypochlorite solution (Dentpro; Ammdent, Chandigarh, India), 17% EDTA solution (Canalarge; Ammdent, Chandigarh, India), SmearClear solution (Sybron Endo, Orange, CA), and Biopure MTAD (Dentsply, Tulsa dental, USA), with Sterile Distilled water (Ranbaxy laboratories Ltd. Mumbai, India) as negative control.

## Materials and Methods

### Methodology

Fifty freshly extracted human single rooted maxillary and mandibular teeth consisting of incisors, canines and premolars were selected for this study. All specimen teeth were randomly divided into four experimental groups and one control group as follows: Group A consisted of distilled water group (negative control group); Group B EDTA; Group C SmearClear; Group D MTAD; and finally Group E used NaOCl irrigation. Each group consisted of ten teeth and was formed on the basis of the type of final irrigation after canal preparation. All samples in a group were treated by a single operator.

Access preparations were made by round diamond burs and patency established by passing a #15 K-file (Dentsply, Maillefer, Ballaigues, Switzerland) beyond the apex of all canals. Working lengths were determined by subtracting 1 mm from the length at which the tip of the file was just visible to the naked eye at the apical foramen. Canals were prepared by the ProTaper rotary system (Dentsply, Maillefer, Ballaigues, Switzerland). Each canal was prepared up to an apical preparation of #F3. Three percent NaOCl (Dentpro, Ammdent, Chandigarh, India) irrigant was used between each subsequent file size in all experimental groups, while sterile distilled water was the sole irrigant in the control group.

To determine the effects of experimental and control solutions as a final rinse on the surface of root canals after instrumentation, the canals were treated with 5 mL of one of the experimental solutions. Initially 1 mL of the solution was used and agitated with a #15 K-file (at a frequency of ≈1.6 Hz i.e. ≈100 times/minute) for 1 minute followed by 4 mL of the irrigant was applied to the canal over 2 minutes. This was conducted identically for all groups (A-E). The irrigating solution was delivered using a 30-gauge side-vented needle (Dentsply Tulsa Dental, Tulsa, OK, USA) passively placed to within the middle third of the root canals.

The total time for final irrigation was 3 minutes for all solutions. The canals were then dried with paper points. Afterwards non-penetrating grooves were made in all specimen teeth at the cementoenamel junction (CEJ) and longitudinally on the buccal and lingual aspects. Crowns of all teeth were removed at the level of the CEJ with a chisel. The decoronated teeth were then longitudinally split into two halves and the half containing the greater part of the apex was selected as the representative sample and coded. Coded samples were then scheduled for scanning electron microscopic (SEM) evaluation.

### SEM Evaluation

**Figure 1:**
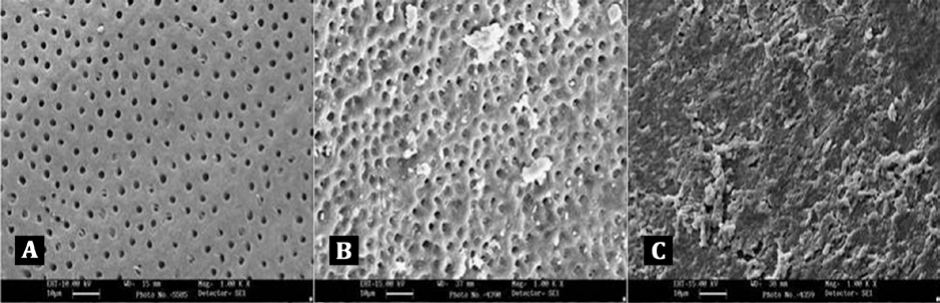
A) Score 1 = no smear layer, no smear layer was detected on the surface of root canal and all tubules were open); B) Score 2 = moderate smear layer, no smear layer on root canal walls but tubules contained debris); C) Score 3 = heavy smear layer, smear layer covered the root canal wall surface and the tubules

Coded samples were dehydrated with ascending concentrations of ethyl alcohol (30-100%) and placed in a desiccator for at least 24h, mounted on metallic stubs, gold sputtered and viewed under a scanning electron microscope (Oxford Instruments, Eynsham, England). The entire length of the sample was divided equally into cervical, middle and apical thirds be evaluated separately. Three photographs at magnifications of 500× and 1000× were taken for each specimen in the coronal third (12 mm from root apex), middle third (08 mm from root apex), and apical third (04 mm from root apex) levels of the root canal system. The images at 1000× magnification were then analyzed for the amount of smear layer present by three independent observers, without knowing which group they were analyzing. The evaluation was repeated twice for the first 10 specimens to ensure intra-examiner consistency. The amount of smear layer remaining on the surface of the root canal and dentinal tubules was scored according to a three score system (Figure 1A-C) developed by Torabinejad et al. [21].

Score 1: no smear layer: no smear layer was detected on the surface of the root canal and all tubules were open. (Figure 1A)

Score 2: moderate smear layer: no smear layer on root canal walls but tubules contained debris. (Figure 1B)

Score 3: heavy smear layer: smear layer covered the root canal wall surface and the tubules. (Figure 1C)

### Statistical Analysis

Owing to the non-parametric nature of the data, non-parametric tests were used for the statistical analysis. The data were analyzed by Kruskal-Wallis and Mann-Whitney U tests. Comparisons were made as follows: pair wise comparison of all groups against the control group at the coronal, middle and apical third level using the Mann-Whitney U test; pair wise intergroup comparison of all experimental groups with each other at the coronal, middle and apical third levels using the Mann-Whitney U test. Intra-group comparison of each group within the coronal, middle and apical third levels using the Kruskal-Wallis test.

## Results

**Figure 2:**
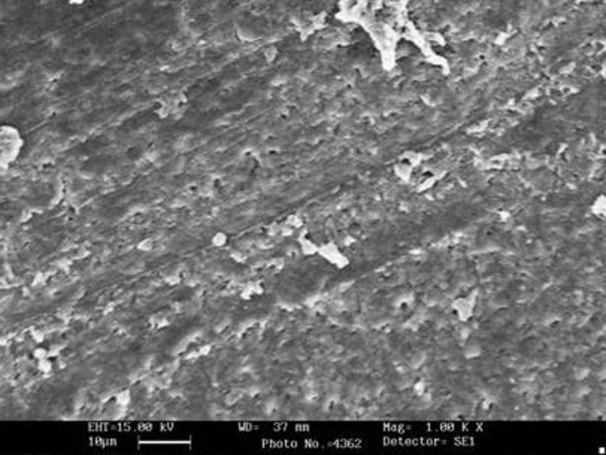
Representative SEM image for group A (control group)

**Figure 3:**
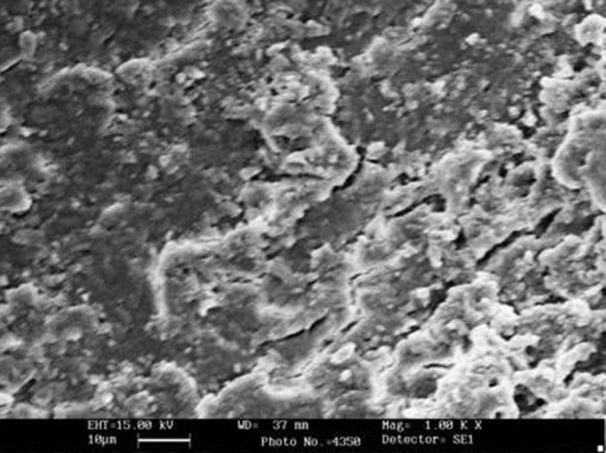
Representative SEM image for group E (NaOCl group)

The examination of the surface of root canal walls in group A (control group) and group E (NaOCl group) showed the presence of a heavy smear layer throughout the entire length of the root canals (Figure 2, Figure 3).

**Figure 4:**
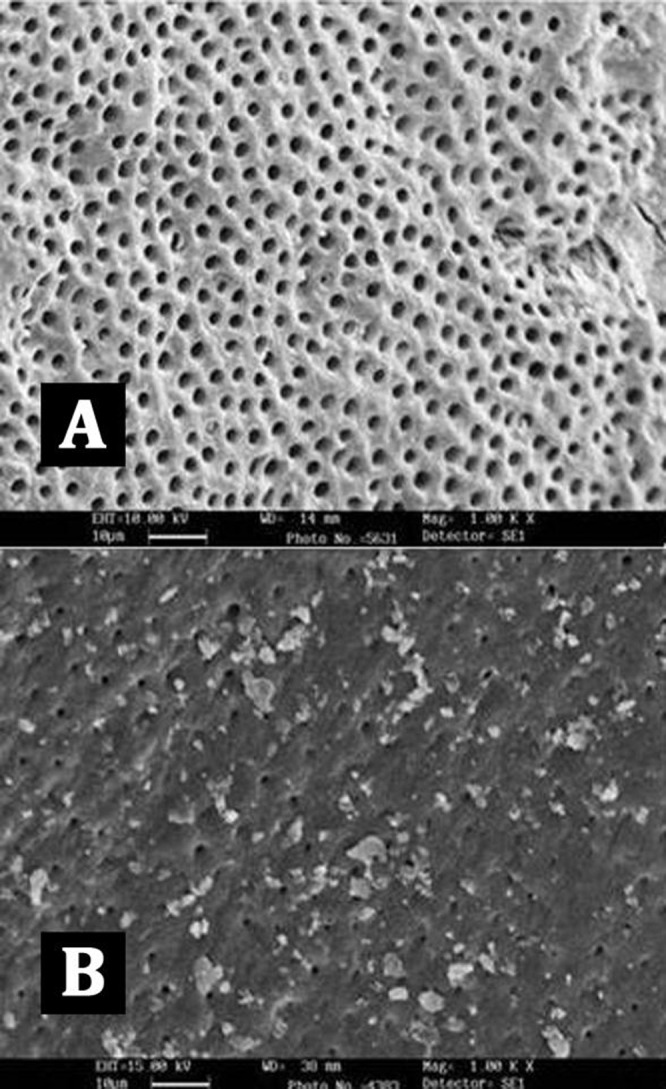
SEM images of group B (EDTA); A) coronal 3rd image; B) apical 3rd

**Figure 5:**
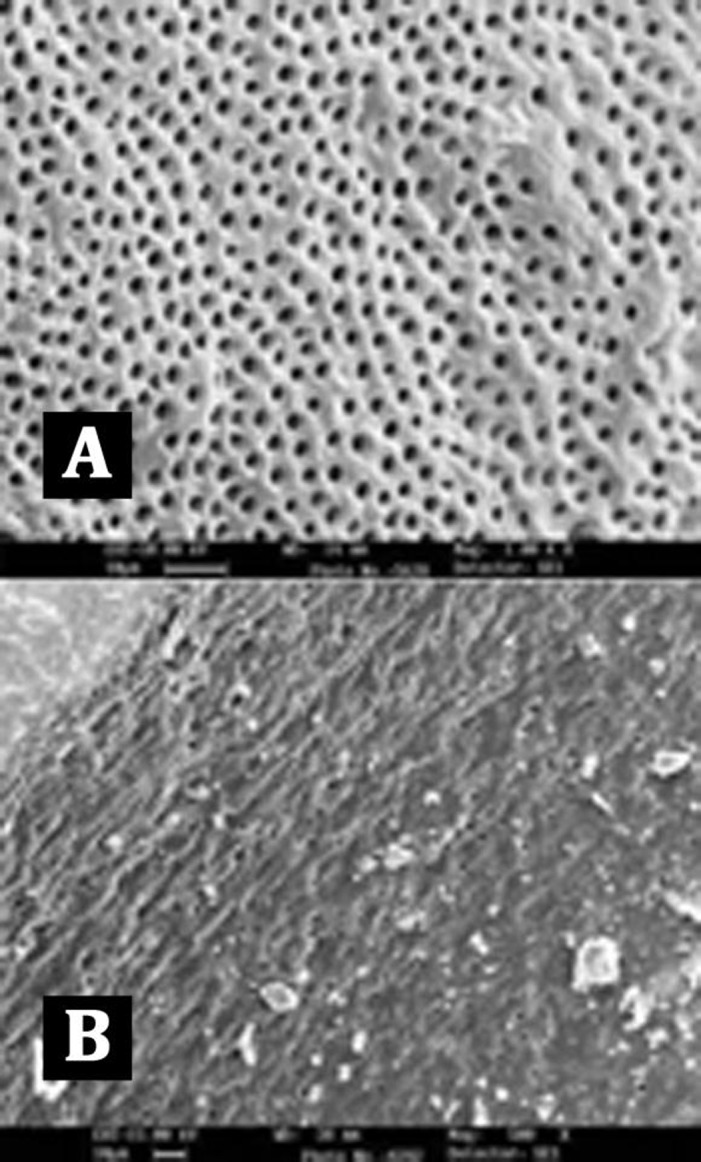
SEM images of group C (SmearClear); A) coronal 3rd image; B) apical 3rd

**Figure 6:**
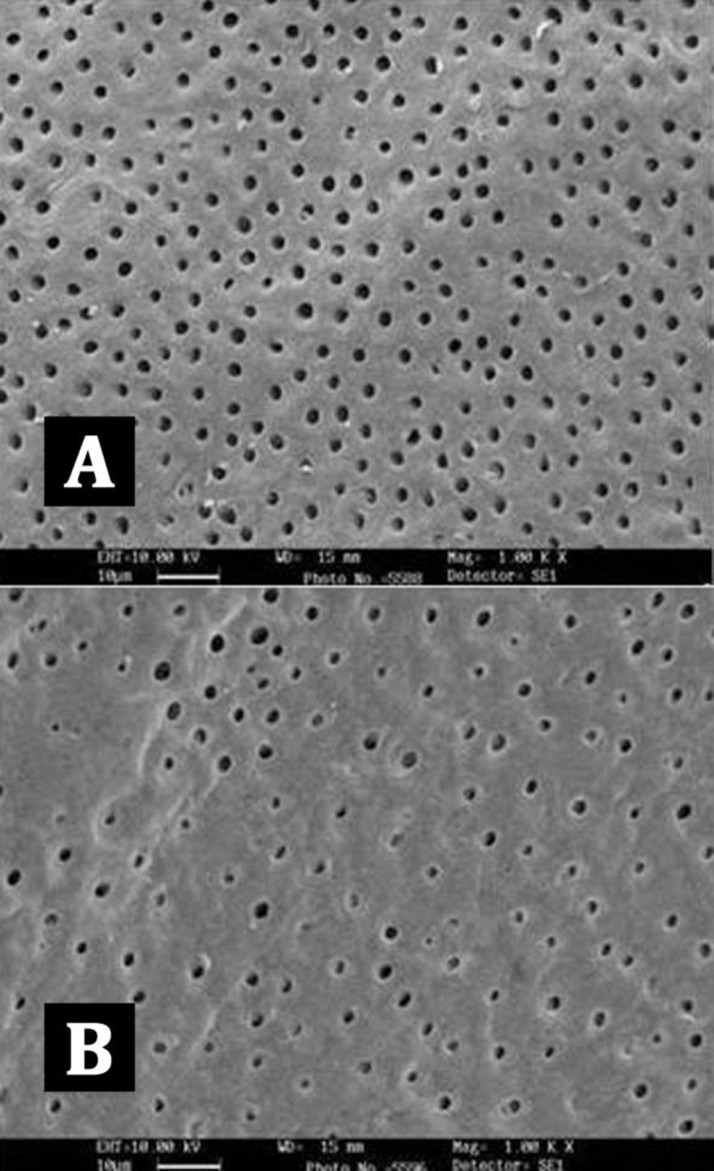
SEM images of group D (Biopure MTAD); A) coronal 3rd; B) apical 3rd

Comparison of the five groups at the coronal, middle and apical thirds showed that the canal walls in group B (EDTA, Figure 4A-B), (SmearClear group, Figure 5A-B), and group D (BioPure MTAD group, Figure 6A-B) were significantly cleaner than in group A (control group) (P<0.001), whereas there was no significant difference in the cleanliness of the canal walls between group E (NaOCl group, Figure 4) and group A (Figure 3) (P>0.05).

Intergroup comparison of groups B, C, and D showed no statistical significant differences in the coronal and middle thirds, but in the apical third the canal surfaces were cleaner in samples from group D (MTAD group) (P<0.05).

Intra-group comparisons of each canal section within each group showed no statistically significant difference in group D (BioPure MTAD group, Figure 6A-B). But in group B (EDTA group, Figure 4A-B) and group C (SmearClear group, Figure 5A-B) the efficacy of the agent was significantly less in the apical third of the samples compared with the coronal third (P<0.05).

## Discussion

In the present study we have used a single common irrigation protocol to compare the smear layer removal efficacies of four different solutions, including two commonly used solutions, 3% sodium hypochlorite and 17% EDTA, and two recently introduced solutions, BioPure MTAD (a mixture of a tetracycline isomer, an acid, and a detergent) and SmearClear (17% EDTA with a cationic (cetrimide) and an anionic surfactant), using distilled water as a negative control.

File agitation was carried out to assist in distributing the irrigant. In this study, sodium hypochlorite alone did not clean the smear layer better than distilled water, proving that irrigation with NaOCl alone is not effective.

All other experimental agents, including 17% EDTA, SmearClear and BioPure MTAD were capable of removing the smear layer from the root canals. Our results demonstrated that the process of smear layer removal was more efficient in the coronal and middle thirds than in the apical third of the canals in group B (EDTA group) and group C (SmearClear group); similar to the results of various other studies [20][22][23]. A larger canal diameter in the coronal and middle thirds exposes the dentin to a higher volume of irrigant, allowing a better flow of the solution and, hence, further improving the efficiency of smear layer removal. The results also demonstrated there was no significant difference between smear layer removal by EDTA and SmearClear in the coronal, middle or apical thirds of the root canal (P<0.05) (Table 1). This further verifies the findings of some previous studies [24][25]. Therefore adding of the surfactants in the SmearClear does not improve its efficacy in removing the smear layer from the root canal. Comparisons between experimental groups showed that in the coronal third and middle third, groups B, C, and D did not differ significantly, but canal surfaces in the apical thirds of group D teeth were significantly cleaner than those in groups B and C (Table 1).

Our results demonstrated that BioPure MTAD was most efficient in removing the smear layer at all levels from the root canal and there was no statistically significant difference in the smear layer removal ability of BioPure MTAD in the coronal, middle or apical thirds (P>0.05) (Table 1). This is in agreement with the results of an earlier study by Torabinejad et al. [21]. Apart from the significant difference between the smear layer removal abilities of BioPure MTAD and EDTA, there are certain disadvantages associated with EDTA, including its destructive effects on the coronal and middle thirds of root dentin and its limited antibacterial effects. In our study, significant erosion of dentinal tubules was also observed in several samples irrigated with 17% EDTA (Figure 4A). On the other hand, Torabinejad et al. [21] reported minimal erosion of intraradicular dentin when NaOCl and MTAD were used in a similar sequence, moreover, BioPure MTAD also possesses antimicrobial activity due to the presence of doxycycline in its formulation.

**Table 1:** Mean smear scores (±SD) in coronal, middle, and apical thirds of the canals in each group

**Group**	**Coronal 3rd**			**Mean(SD)**	**Middle 3rd**			**Mean(SD)**	**Apical 3rd**			**Mean(SD)**	**P-value**
	**1**	**2**	**3**		**1**	**2**	**3**		**1**	**2**	**3**		
**Group A**	0	0	10	3.0±0.0	0	0	10	3.0±0.0	0	0	10	3.0±0.0	1(P>0.05)
**Group B**	8	2	0	1.2±0.42	7	3	0	1.3±0.48	1	6	3	2.2±0.63	0.0058(P<0.01)
**Group C**	8	2	0	1.2±0.42	7	2	1	1.4±0.69	0	7	3	2.3±0.48	0.0026(P<0.01)
**Group D**	9	1	0	1.1±0.31	8	2	0	1.2±0.42	6	4	0	1.4±0.51	0.5092(P>0.05)
**Group E**	0	0	10	3.0±0.0	0	0	10	3.0±0.0	0	0	10	3.0±0.0	1(P>0.05)

## Conclusions

Within the limitation of this in vitro study we can conclude that MTAD is the most effective chemical agent for smear layer removal, especially in the apical third of the root canal, where most formulations of EDTA have proved inefficient. MTAD can be considered as a better alternative to EDTA/NaOCl, as EDTA has no antimicrobial properties and causes dentinal erosion.
